# Phylogenetic and genomic diversity of human bacteremic *Escherichia coli *strains

**DOI:** 10.1186/1471-2164-9-560

**Published:** 2008-11-26

**Authors:** Françoise Jaureguy, Luce Landraud, Virginie Passet, Laure Diancourt, Eric Frapy, Ghislaine Guigon, Etienne Carbonnelle, Olivier Lortholary, Olivier Clermont, Erick Denamur, Bertrand Picard, Xavier Nassif, Sylvain Brisse

**Affiliations:** 1 INSERM UMR570, Faculté de Médecine, Université Paris Descartes, Paris, France; 2 Hôpital Avicenne, AP-HP; UFR Santé, Médecine, Biologie Humaine, Université Paris 13, Bobigny, France; 3 Biodiversity of Emerging Bacterial Pathogens, Institut Pasteur, 28 rue du Dr Roux, 75724 Paris, France; 4 Genotyping of Pathogens and Public Health, Institut Pasteur, 28 rue du Dr Roux, 75724 Paris, France; 5 Faculté de Médecine, Université René Descartes, Hôpital Necker-Enfants Malades, AP-HP, Paris, France; 6 Institut Pasteur, CNRS URA3012, Paris, France; 7 INSERM U722, Faculté de Médecine, Université Paris Diderot, Paris, France

## Abstract

**Background:**

Extraintestinal pathogenic *Escherichia coli *(ExPEC) strains represent a huge public health burden. Knowledge of their clonal diversity and of the association of clones with genomic content and clinical features is a prerequisite to recognize strains with a high invasive potential. In order to provide an unbiased view of the diversity of *E. coli *strains responsible for bacteremia, we studied 161 consecutive isolates from patients with positive blood culture obtained during one year in two French university hospitals. We collected precise clinical information, multilocus sequence typing (MLST) data and virulence gene content for all isolates. A subset representative of the clonal diversity was subjected to comparative genomic hybridization (CGH) using 2,324 amplicons from the flexible gene pool of *E. coli*.

**Results:**

Recombination-insensitive phylogenetic analysis of MLST data in combination with the ECOR collection revealed that bacteremic *E. coli *isolates were highly diverse and distributed into five major lineages, corresponding to the classical *E. coli *phylogroups (A+B1, B2, D and E) and group F, which comprises strains previously assigned to D. Compared to other strains of phylogenetic group B2, strains belonging to MLST-derived clonal complexes (CCs) CC1 and CC4 were associated (P < 0.05) with a urinary origin. In contrast, no CC appeared associated with severe sepsis or unfavorable outcome of the bacteremia. CGH analysis revealed genomic characteristics of the distinct CCs and identified genomic regions associated with CC1 and/or CC4.

**Conclusion:**

Our results demonstrate that human bacteremia strains distribute over the entire span of *E. coli *phylogenetic diversity and that CCs represent important phylogenetic units for pathogenesis and comparative genomics.

## Background

*Escherichia coli *is the most abundant facultative anaerobic bacteria of the human intestinal flora. Whereas *E. coli *usually appears to be a harmless commensal, in other circumstances, *E. coli *strains can be pathogenic to humans and were grouped into various pathotypes [1]. Among these, extraintestinal pathogenic *Escherichia coli *(ExPEC) are responsible for urinary tract, intra-abdominal and soft tissue infections, meningitis, pneumonia and osteomyelitis often associated to bacteremia [2]. Bacteremia represents the tenth major cause of death in developed countries and among Gram-negative bacteria, *Escherichia coli *represents the first cause of bacteremia, with 30% of the total number of bacteremias being due to this pathogen [3].

Among bacteremic isolates, those that are characterized by specific virulence factors (VFs) such as adhesins, capsule, cytotoxins and siderophores are considered as extraintestinal pathogenic *E. coli *(ExPEC) [2], as these VFs are classically described as being necessary to overcome host defenses, invade host tissues and trigger a local inflammatory response [4]. Phylogenetic analyses [5,6] suggested that *E. coli *can be divided into four major groups (A, B1, B2 and D). Generally, human ExPEC strains belong to group B2, and to a lesser extent, group D, whereas commensal strains and less virulent strains belong to A or B1 groups [2,7-9].

Whether *E. coli *virulence factors influence the severity of sepsis, in humans, is not clearly established. In fact, no association between the severity of sepsis and the four phylogenetic groups or other bacterial determinants was evidenced [10]. The difficulty to establish a link between phylogenetic groups on the one hand, and severity of infection in humans on the other hand, can have several causes. In particular, clonal structure within phylogenetic groups and heterogeneity among clonal groups in terms of severity could constitute an important confounding factor. Therefore, it appears important to characterize the clonal structure within phylogenetic groups and its association with clinical features.

Multilocus Sequence Typing (MLST), in which internal portions of multiple housekeeping genes are sequenced to define clonal diversity, has emerged as a powerful tool to describe the genetic structure of bacterial populations [11]. In addition, MLST data allow determining the phylogenetic relationships among deep lineages, providing a complementary view of the population structure [12].

Several sequence-based studies were used to characterize clones or phylogenetic subgroups within *E. coli *[13-21]. Previous analyses of ExPEC were carried out on selected strains [21-25]. However, the identity and distribution of clonal groups in unselected bacteremia episodes have not been investigated systematically, and thus, no unbiased view of the overall diversity and distribution of human bacteremic strains is currently available.

The aims of this work were (i) To define the clonal diversity of all *E. coli *strains isolated from consecutive bacteremia during one year in two major university hospitals in Paris, (ii) To establish the possible association between clonal groups and clinical determinants, and (iii) To characterize the genomic content of the disclosed clonal groups.

## Results

### Nucleotide polymorphism and phylogenetic diversity

The internal portions of eight selected housekeeping genes were sequenced in the 161 isolates, with only two exceptions: three isolates (AVC062, NCK061 and NCK062) were PCR-negative for *trpA*, and three isolates (AVC025, AVC041, NCK011) were PCR-negative for *uidA*. All sequences were aligned without insertion or deletion, with the exception of two isolates (NCK018 and NCK043), which showed a 5-nucleotide deletion in *uidA*, resulting in a frameshift. The proportion of variable sites ranged from 9.3% (*uidA*) to 15.5% (*trpA*) (Table 1). Considering the 4,095 nucleotides of the eight gene portions together, there were 471 (11.5%) variable sites. The nucleotide diversity (π, average number of nucleotide differences per site between two randomly-selected isolates) ranged from 0.0174 (*putP*) to 0.0393 (*trpA*) (Table 1).

**Table 1:** Nucleotide polymorphism found in the eight housekeeping genes used for MLST

Gene	Template size	No. of alleles	No. (%) of polymorphic sites	Ks	Ka	Ka/Ks	π
*dinB*	450	30	59 (13.1)	0.124	0.00197	0.0159	0.0297
*icdA*	516	45	52 (10.1)	0.093	0.0004	0.0043	0.0273
*pabB*	468	32	44 (9.4)	0.075	0.00763	0.102	0.0226
*polB*	450	36	65 (14.4)	0.124	0.00301	0.024	0.0287
*putP*	456	25	43 (9.4)	0.067	0.0015	0.0225	0.0174
*trpA*	561	33	87 (15.5)	0.156	0.00537	0.0345	0.0393
*trpB*	594	25	65 (10.9)	0.133	0.00198	0.0149	0.0299
*uidA*	600	27	56 (9.3)	0.074	0.003	0.0408	0.0183

Ks: No. of synonymous changes per synonymous site. Ka: No. of non-synonymous changes per non-synonymous site.

π: nucleotide diversity.

**Figure 1:**
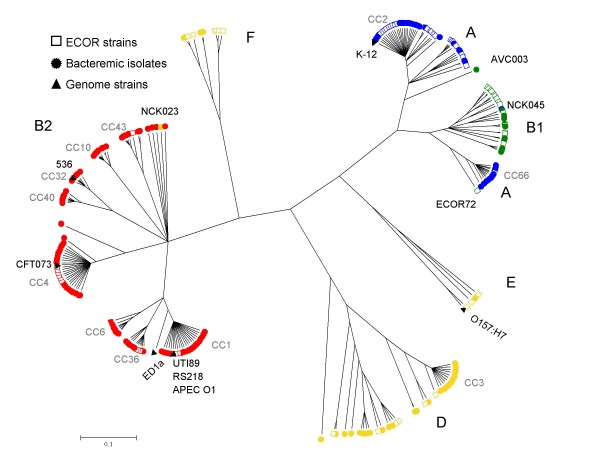
**Phylogenetic diversity**. Phylogenetic analysis performed using ClonalFrame based on the sequence of eight protein-coding genes (4,095 nt in total), of 161 *Escherichia coli *isolates from bacteremia (circles), 67 ECOR strains (open squares) and seven genome reference strains (triangles). Color of strain symbols is according to triplex-PCR grouping into major phylogenetic groups (red, B2; green, B1; yellow, D; blue, A).

**Figure 2:**
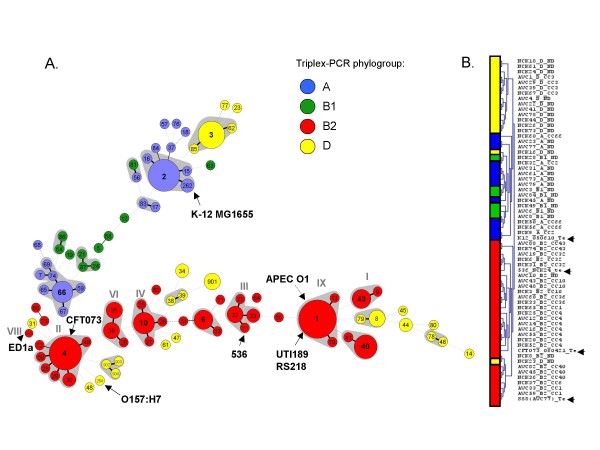
**Cluster analysis of *Escherichia coli *isolates based on allelic profiles (A) and macroarray data (B)**. (A) Minimum spanning tree analysis of the 161 *E. coli *isolates based on allelic profiles at the eight genes *dinB*, *icdA*, *pabB*, *polB*, *putP*, *trpA*, *trpB *and *uidA*. Each circle corresponds to a sequence type (the ST number is given inside the circle), and the size of the circle is related to the number of isolates found with that profile (from 1 for small circles e.g. ST14 to 17 for ST4). Grey zones between some groups of circles indicate that these profiles belong to the same clonal complex (CC). Circles were colored based on triplex-PCR phylogroup assignment. Bold, plain and discontinuous links between circles correspond to one, two or more allelic mismatch(es), respectively; note that links with distance > 2 are not reliable (for example, the spread of group D across the entire graph does not indicate discrepancy of MLST with triplex PCR). Grey roman numbers above some CCs correspond to B2 subgroups as defined in a previous study [21]. The position of strain APEC O1 was added manually; it corresponds to a single locus variant of ST1. (B) Classification of 60 bacteremic isolates using macroarray analysis results. Reference strains are indicated by black arrows on the right. Note that two apparently misplaced isolates (NCK023 and NCK016) belong to groups B2 and E, respectively, based on MLST genes.

*E. coli *strains are generally considered to be grouped into the four major phylogenetic groups A, B1, B2 and D [5,6], although some strains may belong to additional groups [19,26]. We characterized all isolates by the triplex PCR method, designed to typify strains into four categories equated to the four major groups [27]. Based on this PCR scheme, the number of bacteremic isolates classified into groups A, B1, B2 and D were 33 (20%), 11 (7%), 80 (50%) and 37 (23%), respectively. To determine precisely the phylogenetic diversity of the 161 bacteremic isolates, we sequenced the eight gene portions in strains of the ECOR collection and performed a joint phylogenetic analysis, together with seven genome reference strains (Figure 1). The results highlighted the strong phylogenetic clustering of *E. coli *strains into five sharply separated branches. Based on triplex PCR results and ECOR strains, these branches could be equated to groups B2, A+B1, D, E and a group, which we refer to as group F, comprising strains assigned to group D based on triplex PCR. Of note, when genetic distances among strains were considered, the B2 branch was more heterogeneous than the branch comprising strains of groups A and B1 together. Group A appeared paraphyletic, as B1 strains were nested within the diversity of A strains. A few confirmed inconsistencies (for isolates NCK23, NCK45 and AVC003, Figure 2) were found between triplex-PCR and sequence-based phylogenetic relationships, as also reported by others [20,26]. Remarkably, the bacteremic isolates were distributed into the five major branches, clearly demonstrating that they do not represent a restricted subset of *E. coli *strains.

### Genotype diversity and identification of clonal complexes

The 161 clinical isolates were distributed into 87 sequence types (ST), seven of which comprised five or more strains, representing 38% of the strains in total (Additional file 1). The most frequent ST encountered was ST1 (ST78 or ST79 in Mark Achtman's MLST scheme; please see Additional file 2 for known correspondence of STs in our scheme and Mark Achtman's one), with 15 isolates. Sixty-nine STs corresponded to a single isolate, and Simpson's diversity index was 97.6%, underlying the diversity of bacteremic *E. coli *isolates revealed by MLST. Of note, frequent STs mostly belonged to groups A and B2, resulting in a lower Simpson's index of diversity of groups A (87.7%) and B2 (92.9%), compared in particular to group B1, were all sampled isolates were distinct (Simpson's index, 100%). There was no clear difference in genotype diversity between the two hospital centers, and frequent STs were recovered from each of them.

Clonal families within bacterial populations in which homologous recombination occurs, as is the case for *E. coli *[19,28], are best identified based on allelic profile comparisons, as the collapsing of nucleotide polymorphisms into allelic numbers is less sensitive to the disturbing impact of recombination [12]. As determined using eBURST and a minimum spanning tree (MStree) analysis (Figure 2), the 87 STs were grouped into 19 clonal complexes (CC), i.e. groups of closely related STs differing by no more than one allele from another member of the group. Eleven major CCs (CC1 to CC4, CC6, CC10, CC32, CC36, CC40, CC43 and CC66) comprised five or more isolates. Based on available complete genome sequences and ECOR strains, correspondence of CCs with a previous classification [21] was established (Figure 2). Interestingly, no member of subgroup VIII (ST149) [21] was found among the bacteremic clinical isolates. Strains of this clone, represented by the ED1a genome strain (Figure 2), have been reported as human-specific and strictly commensal [29]. Each individual CC probably has a single ancestor for most parts of its genome, and CCs can therefore be equated to clones, as it is very likely that strains sharing seven out of eight alleles have a common descent. Although large allelic profile distances separated most CCs, the links among clones disclosed by MStree analysis (Figure 2A) were consistent with phylogenetic group assignments. For example, CCs of group B2 generally shared more alleles in common than with STs of other groups.

### Association of clonal complexes with clinical determinants

In a previous work, differences were found among phylogenetic groups with respect to the primary source of bacteremia [30]. B2 was significantly associated with urosepsis and immunocompetent hosts, whereas non-B2 isolates were associated with non-urinary tract origins and immunocompromised hosts. Here, we tested the hypothesis of a possible association of particular clones (the above described 11 major clones), even within a given phylogenetic group, with clinical determinants (Additional file 1).

Based on a logistic regression model analysis, we did not find statistical evidence for an association of CCs with a severe sepsis or a defavorable outcome. However, the two major clones of group B2 (CC1 and CC4) were significantly associated with urinary tract as a source of bacteremia (Table 2), in comparison to other B2 genotypes. There were 12 (70%) urosepsis cases out of 17 isolates of CC1 (p = 0.017), and 11 (65%) urosepsis cases out of 17 isolates of CC4 (p = 0.041). In contrast, in the 46 remaining B2 isolates, there were only 14 (30%) urosepsis cases. Conversely, B2 clone CC32 was significantly associated with a non urinary tract origin as primary source of infection (p = 0.023). The other clones of group B2 showed mixed origins with few urosepsis cases (Table 2). A number of other characteristics of particular clones compared to other B2 isolates were statistically supported: CC36 was associated with female gender (p = 0.005), although it was not associated with urinary origin; CC32 was found only in males (p = 0.029), CC40 was associated with community-acquired infection (p = 0.048) and CC6 tended to be associated with diabetes mellitus (p = 0.059). These results may indicate heterogeneity of biological characteristics among CCs of phylogenetic group B2. Similarly, among phylogenetic group D isolates, CC3 was significantly associated with a digestive tract origin of the infection (p = 0.038). Among group A isolates, isolates of CC66 were significantly associated with renal failure (p = 0.049), and CC2 isolates were weakly associated with neoplasia (p = 0.069).

**Table 2:** Relationships between clinical determinants and the 11 major clonal complexes

		A		B2								D
	All	CC2 (n = 13)	CC66 ((n = 11)	CC1 (n = 17)	CC4 (n = 17)	CC36 (n = 7)	CC6 (n = 5)	CC32 (n = 6)	CC10 (n = 8)	CC40 (n = 7)	CC43 (n = 5)	CC3 (n = 10)

Age ≥ 65 years old	82	5	7	9	6	3	3	2	4	3	3	6
Male gender	84	8	4	10	8	0**	3	6**	4	4	1	5
Diabetes mellitus	33	2	2	5	1	1	3*	1	1	1	1	1
Neoplasia	30	5*	3	3	1	2	0	1	2	2	0	2
Hematological malignancy	32	1	3	3	2	2	2	0	2	0	1	4
HIV infection	5	1	0	0	0	0	0	0	0	0	0	1
Renal failure	33	1	5**	1	5	1	1	1	1	1	2	3
Immune status												
Immunocompetent	102	7	7	14	10	3	3	5	6	7**	2	4
Immunocompromised	59	6	4	3	7	4	2	1	2	0	3	6
Origin of infection												
Community-acquired	99	7	6	12	12	4	3	4	6	5	2	5
Nosocomial	62	6	5	5	5	3	2	2	2	2	3	5
Source of bacteremia												
Urinary tract	66	4	3	12**	11**	4	2	0*	4	3	1	1**
Digestive tract	59	7	4	5	3	2	3	2	2	1	3	7**
Others and unknown	36	2	4	0**	3	1	0	4**	2	3	1	2
Sepsis stage												
Sepsis	131	12	9	15	13	7	5	4	8	4	4	6
Severe sepsis	12	1	0	0	2	0	0	1	0	2	1	1
Septic shock	18	0	2	2	2	0	0	1	0	1	0	3
Died	16	0	0	1	1	1	0	2	0	2	1	2

Data are displayed as number of isolates. CC: clonal complex.

* p < 0.1 for considered CC vs. all other isolates.

** p < 0.05 for considered CC vs. all other isolates.

### Virulence factor content of clonal complexes

Virulence factor (VF) distribution is known to differ among the four phylogenetic groups [5-7]. In our previous study, isolates of group B2 were characterized by a high number of VFs, whereas those belonging to groups A and D exhibited lower numbers [30]. Here, we compared at the clone level the presence of nine genes associated with ExPEC virulence, and of the *svg *ORF, recently described as specific for a highly virulent B2 subgroup [23] (Additional file 1). As expected, we found a strong association between CCs and VF content, particularly among B2 isolates (Table 3). For example, clones CC1 and CC4 differed significantly by the number of isolates containing *hlyC, cnf *and *sfa *(low in CC1 but high in CC4). In addition, *iucC *was significantly associated with CC1 and CC4 isolates, compared to the remaining B2 isolates. The absence of the adhesin *papGII *was statistically associated with B2-CC32, B2-CC10 and B2-CC40, compared to the other B2 isolates. In addition, B2-CC43 and B2-CC6 were almost devoid of the investigated VFs, with statistical support for *papC*, *sfa*, *hlyC*, *cnf1 *and *iucC *(p < 0.05). Finally, all isolates of ST1 (the central genotype of CC1) except one were positive upon *svg *PCR, consistent with previous results [23]. Only one non-ST1 isolate (ST41, member of CC40) was also positive for *svg*.

**Table 3:** Virulence factor content of the 11 major clonal complexes

		A			B2									D
Bacterial determinants	All	All A	CC2 (n = 13)	CC66 (n = 11)	All B2	CC1 (n = 17)	CC4 (n = 17)	CC36 (n = 7)	CC6 (n = 5)	CC32 (n = 6)	CC10 (n = 8)	CC40 (n = 7)	CC43 (n = 5)	CC3 (n = 10)
papC	79	9	4	3	60	15*	13*	7*	2	5*	8*	5	1**	3
papG II	47	2	1	0*	36	15*	12*	4	2	0**	0**	0**	1	3
papG III	27	0	0	0	27	0	4	3§	0	5*	7*	5*	0	0
Sfa	47	1	0*	1	46	2**	16*	7*	1	4§	8*	5*	0**	0*
hlyC	51	2	0*	1	48	3**	15*	7*	0**	5*	7*	7*	1	0*
cnf1	48	1	0*	1	47	2§**	15*	7*	0**	5*	8*	7*	0**	0*
iucC	106	23	9	10§	52	15§**	15§**	5	5	1*	0*	3	4	7
iron	93	15	4§	7	63	15*	16*	7*	1**	4	8*	7*	1**	3§
fyuA	125	19	8	8	80	17*	17*	7	5	6	8	7	5	4*

Data are displayed as number of isolates. CC, clonal complex; *papC*, P fimbriae; *papGII*, adhesin PapG class II; *papGIII*, adhesin PapG class III; *sfa*, S fimbriae; *hlyC*, hemolysinC; *cnf1*, cytotoxic necrotizing factor; *iucC*, iron uptake system (IUS) aerobactin; *fyuA*, IUS yersiniabactin; *iroN*, IUS salmochelin.

§p < 0.1 for considered CC vs. all other isolates.

* p < 0.05 for considered CC vs. all other isolates.

** p < 0.05 for considered CC vs. all other B2 isolates.

### Flexible gene pool content of bacteremic isolates

In order to determine the genomic characteristics of clones and to identify possible associations between genetic factors and clinical features, we designed a macroarray composed of 2,324 probes and analyzed the genome content of a subset of 60 bacteremic isolates selected to be representative of the diversity of genotypes (Additional file 1). Generally, the DNA array hybridization results were in accordance with housekeeping genes-based phylogeny and genotypic (ST) classification. Cluster analysis of hybridization data grouped all B2 isolates in a single major group and other phylogroups were also recovered with few exceptions (Figure 2B). Interestingly, subgroups recovered by cluster analysis of DNA array data corresponded to clonal complexes with a single exception (NCK065-CC1, Figure 2B), showing that CCs are characterized by a specific genomic content. In addition, DNA array data were fully consistent with the above-described virulence factor PCR results.

**Figure 3:**
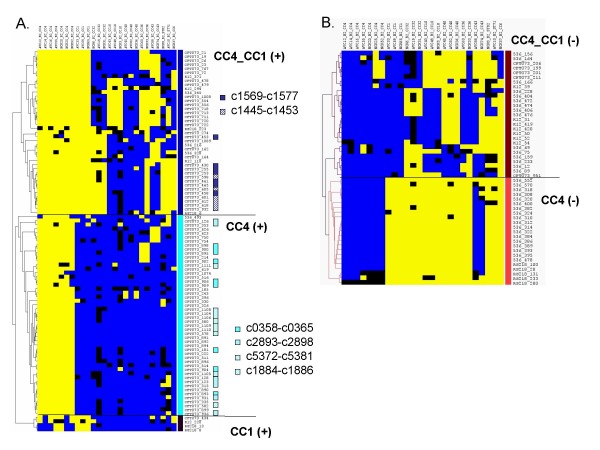
**Open reading frames that distinguish the two clonal complexes CC1 and CC4 from other *Escherichia coli *isolates**. Pattern of presence (yellow) or absence (blue) of ORFs, which presence was positively (A) or negatively (B) associated with B2 isolates of CC1 and/or CC4 isolates, as compared with non-CC1, non-CC4 B2 isolates. Each row corresponds to one ORF; columns represent the 26 assayed B2 isolates grouped by clonal complex. Blue: absent; yellow: present; black: inconclusive. Note that ORFs are clustered vertically using software TMeV based on their pattern of presence among isolates. Five groups can be distinguished based on presence/absence in CC1 and/or CC4, as commented on the right. In panel A), colored squares on the right indicate that ORFs belong to genomic clusters (see text), as indicated.

Because the strongest link of clonal diversity with clinical features within major phylogenetic groups was the association of two B2 clones (CC1 and CC4) with urinary tract origin, we concentrated our analysis on the genomic particularities of CC1 and CC4. We identified 145 (6%) probes that were distributed differently in CC1 and/or CC4 when compared to other B2 isolates (Figure 3, Additional file 3), as determined by the template matching clustering approach [31].

Among probes that reacted positively with both CC1 and CC4 but not with other B2 isolates, were two clusters of six and eight sequences, respectively (c1445-c1453 and c1569-c1577) (Fisher exact test, p < 0.05) (Figure 3). These prophage sequences are included in previously described genomics islands [32,33] in the CFT073 chromosome, respectively φ-CFT073-*potB *and PAI-CFT073-*icdA*. Consistently, these sequences were also present in the O45:K1 strain S88 (ST1, CC1), a meningitis-causing strain that is closely related [34] to *E. coli *K1 RS218 strain (ST1, CC1). The two groups of phage-associated sequences were not restricted to CC1-CC4 B2 isolates (present in strain 536 and B2-CC32, Figure 3) and also detected in some isolates of groups A, B1 and D.

Genomic regions that were specific for CC4 were also identified. Isolates of CC4 were characterized, compared to other B2 isolates, by the presence of three operons composed respectively of three (c1884-c1886), six (c2893-c2898) and seven genes (c5372-c5381) (p < 0.001, Fisher exact test), encoding putative proteins of unknown function. Consistently, these genes were present in the genome of CFT073 (ST4, CC4), but not in the genomes of strains 536 (ST261, CC32) and S88 (ST1, CC1). Moreover, these operons were detected in none of the non-B2 isolates. In addition, a large DNA region from CFT073 (c0358-c0365) was detected more significantly in CC4 isolates than in other B2 isolates (p = 0.01 Fisher exact test, present also in B2-CC36), and all isolates of groups A, B and D were negative for this DNA region (Figure 3 and Additional file 3). This region comprised three genes (c0361-c0363) encoding two putative membrane export proteins and a putative RTX exotoxin A. Regarding CC1-associated genes, only two bacteriophage proteins described in RS218 (ST1, CC1) genome, one Q homolog phage protein and a K-12 MG1655 hypothetical protein (*ydfC*), were found. Conversely, two groups of sequences were always absent from CC1 and CC4 but present in most other B2 isolates (Figure 3; p < 0.05, Fisher exact test).

## Discussion

We analyzed the phylogenetic diversity of an unselected set of 161 consecutive bacteremic *E. coli *isolates, and compared the genomic content of a representative subset of genotypes in order to investigate the link between clonal diversity, gene content and clinical correlates of bacteremia.

Bacteremic isolates were distributed across the entire span of *E. coli *phylogenetic diversity. The amount of polymorphism found herein is consistent with values reported in *E. coli*, based on other housekeeping genes [18,19,35,36]. Amounts of polymorphism among bacteremia isolates were only slightly lower than a recent study of 185 isolates from freshwater beaches [18]. For example, the authors found 49 *uidA *alleles with 12% polymorphic sites, whereas we found 27 *uidA *alleles and 9.3% polymorphic sites (note that our collection of bacteremic isolates included only 7% of isolates of group B1, whereas this group represented 56% of isolates from the environment [18]). Consequently, our results clearly indicate that human *E. coli *bacteremia strains are genetically highly diverse. Of note, avian pathogenic *E. coli *strains (APEC) overlap only partly with the human ExPEC population, with some potentially zoonotic clonal groups being found both in humans and birds, while other APEC clonal groups are rare or absent among human ExPEC [37-39]. Thus, total ExPEC diversity may exceed the large diversity of human bacteremic isolates alone.

Although it was not the main purpose of this study, we noted that our combined analysis of bacteremic isolates and of ECOR reference strains using ClonalFrame revealed the existence of sharp demarcations among deep branches, suggesting a much stronger structure within the *E. coli *species than previously disclosed based on recombination-sensitive phylogenetic analyses, such as the neighbor-joining method. So far, methods that detect homologous recombination events and account for these in phylogenetic reconstruction have not been used widely in *E. coli*, and it is likely that phylogenetic group demarcation may have been problematic until now given the effects of homologous recombination, which tends to homogenize *E. coli *diversity and blur the neat borders that may delineate independently evolving phylogenetic lineages [19,26]. In addition, classification into only four major groups appears to be an oversimplification of a more complex reality. The five major branches disclosed herein do not correspond totally to previous group definitions, as groups A and B1 were not well separated, and as branch F could not be equated to any previously described group. These results challenge the classical view of the internal phylogenetic structure of *E. coli*, in agreement with recent studies [18,19].

Our initial hypothesis was that clones, rather than entire phylogenetic groups, may be more relevant natural entities to establish an association of genotype with phenotype, including clinical correlates of bacteremic isolates. In order to define groups of closely related genotypes, we used allelic profile-based comparisons, rather than nucleotide sequence-based analysis [16,21,23]. Because the later approach is sensitive to homologous recombination, analysis of allelic profiles is preferred for clone definition in many bacterial species [12,40]. Among *E. coli *bacteremic isolates, a number of CCs, which can be equated to clones, could thus be defined. Interestingly, the central genotype of CCs, inferred to represent the founder of the clone, generally had a higher frequency than its variants, consistent with currently ongoing or recent expansion of the clones [12]. For the purposes of classification, it is noteworthy that most CCs were separated by large allelic profile distances (four or five mismatches), highlighting the neat demarcation among them. Phylogenetic group B2 appeared to be the most strongly structured group, as most isolates were grouped into nine CCs (Figure 2).

We explored the hypothesis that clinical correlates and distribution of particular genes would be associated with specific clonal backgrounds, rather than being distributed widely across *E. coli *diversity. Because horizontal gene transfer occurs among *E. coli *strains, virulence genes that confer specific advantages e.g. for urinary tract infection can be distributed in various genomic backgrounds. However, association of specific genes with particular genomic backgrounds (clonal groups) can be retained, at least in the short term, by predominantly vertical transmission e.g. for genes that are not harbored on mobile elements. In this case, knowledge of the clonal background would have predictive value regarding gene content and corresponding phenotypes [41].

Consistent with our initial hypothesis that particular clones may exhibit clinically relevant features, several correlations were established between some CCs and clinical data. Most notably, CC1 and CC4 were clearly associated with isolates responsible for urosepsis; these CCs correspond to previously-described subgroups II (strain CFT073) and IX (strain RS218), corresponding to strains responsible of urosepsis and of neonatal meningitis, respectively [21]. Hence, the previously held association of B2 as a whole with urosepsis may be an oversimplification due to the strong contribution of CC1 and CC4 to isolates of this group, and may thus not be valid for all B2 strains. Interestingly, we detected by DNA array analysis, two specific gene clusters associated with CC1 and CC4. These sequences, which correspond to cryptic prophages, were previously described as being located in two consecutive genomic islands [32]. Consistent with our results, one of these clusters, PAI-CFT073-*icdA*, was described as being more frequent among pyelonephritis isolates [32]. Likewise, a specific DNA region encoding to a putative RTX protein was significantly associated to CC4 isolates. A first RTX-toxin described in *E. coli*, hemolysin A, has been clearly associated with pyelonephritis and implicated in inflammation during urinary tract infection [42,43]. Some UPEC strains including CFT073 harbor two operons of this toxin, and the specific role of each copy remains unclear [33,44]. Whether the new putative RTX toxin constitutes an advantage to CC4 isolates and could explain their specific urovirulence remains to be determined.

As assessed by PCR and DNA array hybridization, clonal complexes were characterized by specific gene content. For example, isolates belonging to CC4 exhibited significantly more VFs (*sfa, cnf1, hlyC*) than CC1 isolates, and ORF *svg *was associated with CC1. In addition, the pattern of gene content variation (Figure 3) was highly concordant with clonal complexes, clearly illustrating that genomic background as assessed by MLST and gene content are strongly correlated. This pattern is consistent with the well-established mechanisms of deletion or acquisition of entire PAIs [45-50]. In order to get clues into the possible biological and clinical significance of gene content differences among clones, it will be necessary to combine functional studies with the analysis of *E. coli *isolates from other sources. For example, comparing the gene content of bacteremic and commensal isolates within a single clone should provide insights into microevolutionary events leading to increased or decreased pathogenic potential.

Our results confirmed that most of the usually recognized extra-intestinal VFs (e.g. *pap, sfa, hly*) were concentrated in the virulent CCs, particularly those belonging to phylogenetic group B2. In contrast, others VFs, for example those related to the plasmid-encoded iron uptake system (e.g. *iucC, iroN*), were more broadly distributed [45,51]. Therefore, for highly mobile genetic elements (e.g. plasmids), the association with clonal background may not be retained.

## Conclusion

In conclusion, we characterized the clonal diversity of all consecutive isolates responsible for bacteremia during one year in two hospitals, revealing for the first time their distribution into phylogenetic groups and clonal groups in an unbiased way. We demonstrated the existence of differences in gene content or clinical parameters among some of the major clonal families identified. Although they should be confirmed on larger populations to be firmly established, these results confirm that particular STs, CCs or phylogenetic subgroups, rather than the more inclusive higher categories known as major phylogenetic groups, will be relevant units of pathogenicity. However, the heterogeneity of gene content within clones, although limited, may be highly significant clinically, which in turn suggests that finer phylogenetic subdivisions within CCs may also differ in clinically important characteristics. The failure to associate CCs with severity of clinical outcome may also indicate that the characteristics of the host play a dominant role.

## Methods

### Bacterial strains

We studied 161 well-characterized *E. coli *isolates from bacteremia; this set of isolates (Additional file 1) has been previously described [30]. Briefly, isolates were collected between December 2002 and December 2003 and correspond to all consecutive episodes of *E. coli *bacteremia in two major university hospitals in Paris. Epidemiological (age, sex gender), clinical (community/nosocomial acquired infection, immune status, underlying diseases, primary source of infection, severity sepsis scoring and outcome), and laboratory data for each episode were recorded in an anonymous computer database in accordance with French law. We determined the primary source of bacteremia by clinical and radiological presentation and/or by evidence, based on antibiogram and/or serotyping, of an identical strain isolated from blood and other body sites culture [30]. When the primary source of infection was not found, the origin of infection was assigned to the digestive tract. All the above 161 isolates are listed in **supplementary Table 1**. The ECOR collection of 72 reference strains [52] was included for phylogenetic comparisons; five ECOR strains could not be sequenced at all eight genes (see below) and were removed from the analysis: ECOR5 (A), ECOR6 (A), ECOR9 (A), ECOR13 (A) and ECOR64 (B2). In addition, seven strains for which the complete genome sequence is available (K-12 MG1655, O157:H7-EDL933, 536, CFT073, UTI89, RS218) or underway (ED1a, ColiScope project, ) were used.

### Triplex-PCR for tentative group assignment

A triplex-PCR method [27] was used to tentatively assign the isolates to the four major *E. coli *phylogenetic groups A, B1, B2 and D.

### Multilocus Sequence Typing

Primer pairs were designed for PCR amplification and sequencing of internal portions of eight housekeeping genes (Table 4). Selected genes included *dinB*, *icdA, pabB, polB, putP, trpA *and *trpB*, previously used for phylogenetic analysis of *E. coli/Shigella *strains [53-55]. New PCR primers were designed in internal portions of the genes based on previously obtained sequences, in order to amplify target regions of approximately 500 – 600 bp (Table 4). These seven genes represent six distinct loci on the *E. coli *chromosome, as *trpA *and *trpB *are located in the same operon. To increase the number of loci to seven, we added gene *uidA*, which is used for *E. coli *MLST by the group of Tom Whittam [20]. Universal sequencing primer sequences were added to the 5' end of the PCR primers. All PCR products were thus sequenced using the same two sequencing primers (Table 4). Further details on this MLST scheme can be found at .

**Table 4:** Primers used for MLST

Locus	Function	Forward primer	Reverse primer	Location (a)
*dinB*	DNA polymerase	5'-GTT TTC CCA GTC ACG ACG TTG TAT GAG AGG TGA GCA ATG CGT A-3'	5'-TTG TGA GCG GAT AAC AAT TTC CGT AGC CCC ATC GCT TCC AG-3'	282,284 – 282,734
*icdA*	Isocitrate dehydrogenase	5'-GTT TTC CCA GTC ACG ACG TTG TAA TTC GCT TCC CGG AAC ATT G-3'	5'-TTG TGA GCG GAT AAC AAT TTC ATG ATC GCG TCA CCA AAY TC-3'	1,118,658 – 1,187,173
*pabB*	p-aminobenzoate synthase	5'-GTT TTC CCA GTC ACG ACG TTG TAA ATC CAA TAT GAC CCG CGA G-3'	5'-TTG TGA GCG GAT AAC AAT TTC GGT TCC AGT TCG TCG ATA AT-3'	1,807,273 – 1,807,740
*polB*	Polymerase PolII	5'-GTT TTC CCA GTC ACG ACG TTG TAG GCG GCT ATG TGA TGG ATT C-3'	5'-TTG TGA GCG GAT AAC AAT TTC GGT TGG CAT CAG AAA ACG GC-3'	65,220 – 64, 773
*putP*	Proline permease	5'-GTT TTC CCA GTC ACG ACG TTG TAC TGT TTA ACC CGT GGA TTG C-3'	5'-TTG TGA GCG GAT AAC AAT TTC GCA TCG GCC TCG GCA AAG CG-3'	1,074,708 – 1,075,163
*trpA*	Tryptophan synthase subunit A	5'-GTT TTC CCA GTC ACG ACG TTG TAG CTA CGA ATC TCT GTT TGC C-3'	5'-TTG TGA GCG GAT AAC AAT TTC GCT TTC ATC GGT TGT ACA AA-3'	1,338,658 – 1,338,098
*trpB*	Tryptophan synthase subunit B	5'-GTT TTC CCA GTC ACG ACG TTG TAC ACT ATA TGC TGG GCA CCG C-3'	5'-TTG TGA GCG GAT AAC AAT TTC CCT CGT GCT TTC AAA ATA TC-3'	1,339,430 – 1,338,837
*uidA*	Beta-glucuronidase	5'-GTT TTC CCA GTC ACG ACG TTG TAC ATT ACG GCA AAG TGT GGG TCA AT-3'	5'-TTG TGA GCG GAT AAC AAT TTC CCA TCA GCA CGT TAT CGA ATC CTT-3'	1,615,010 – 1,614,411
OF	Sequencing primer (b)	5'-GTT TTC CCA GTC ACG ACG TTG T-3'		
OR	Sequencing primer (b)	5'-TTG TGA GCG GAT AAC AAT TTC-3'		

(a) Based on *Escherichia coli *strain 536, complete genome, GenBank accession CP000247.

(b) PCR primers have the corresponding universal sequence in 5'.

Nucleotide sequences were obtained using Big Dye version 3.1 chemistry on ABI 3100 or 3730 apparatuses. In order to eliminate the risk of sample mix-up, PCR and sequencing were performed using a molecular biology robot (RoboAmp 4200-PE; MWG Biotech, Courtaboeuf, France). Sequence chromatograms were edited and stored using BioNumerics version 4.5 (Applied-Maths, St. Maartens-Latem, Belgium). All nucleotides within the consensus sequence template were supported by at least two sequence chromatograms. A different allele number was given to each distinct sequence within a locus, and a distinct sequence type (ST) number was attributed to each distinct combination of alleles. Null alleles corresponding to negative PCR amplification were considered as alignment gaps in phylogenetic analyses and as allele '999' in profile-based analyses. Isolates were grouped into clonal complexes (CCs) by eBURST, if they differed at no more than 1 locus from at least one other member of the group [56]. Founder genotypes of CCs were defined as the ST of the CC with the highest number of neighboring STs (single locus variants). Nucleotide diversity was calculated using DNAsp version 4 [57]. Minimum spanning tree analysis was performed using BioNumerics version 5.10. MEGA [58] was used to draw the consensus phylogenetic tree obtained using ClonalFrame [59] after 100,000 iterations, including 50,000 burn-in.

### Sequences

Allele sequences and STs are available on Institut Pasteur's MLST web site at .

### DNA macroarrays

A DNA macroarray was adapted from a previously described array [21]. This *E. coli *pathoarray was developed with the aim to contain a large *E. coli *flexible gene pool. DNA probes were selected using the genome sequence (GenBank accession CP000247) of the O6:K15:H31 uropathogenic *E. coli *strain 536 [60], the sequence (GenBank accession AE014075) of the O6:K2:H1 uropathogenic *E. coli *strain CFT073 [33], and *E. coli *K-12 (MG1655, GenBank accession U00096) [61]. DNA sequences showing an homology above 95% within the three genomes were excluded. In addition, specific sequences of the meningitis-associated strain RS218 [62], i.e. sequences with less that 95% homology in the above three strains, were included. The genome sequence of strain RS218 was only partially available at  at the time of membrane conception.

Amplicons were designed to correspond to annotated ORFs. Additional file 4 describes the composition of the membrane. When the size of an ORF was less than 800 bp the entire sequence corresponding to the ORF was selected. ORF above 1 kb were represented on the membrane by at least 2 amplicons. In addition sequences of intergenic regions which were not present in all three genomes were amplified and represented on the membrane if above 500 bp. 2,324 specific genomic DNA fragments corresponding to 2,196 ORFs and 91 intergenic regions (IRs) were spotted on the membrane. Fourteen additional sequences were added in the DNA array. These sequences corresponded to specific virulence-associated ExPEC genes characterized in recent studies but not represented after comparative genomic analysis of the above strains.

The amplicons were spotted in duplicate by a robot (Eurogentec, Seraing, Belgium) on nylon membranes and fixed by alkali treatment. Positive (186 spots containing 16S rDNA and genomic DNA of CFT073 strain) and negative (15 spots containing both human and mouse gene DNA and 14 spots with absence of DNA) control spots were used for normalization.

### Genomic DNA extraction, labeling and macroarray hybridization

Bacteria were grown overnight at 37°C in aerobic condition on agar plate. Genomic DNA was isolated using the DNA Wizard Genomic DNA Purification Kit (Promega, Madison, USA). Genomic DNA extract (100 ng to 500 ng) were labelled with 50 mCi of [α^33^P] dCTP (Amersham Biosciences, Orsay, France), using the Rediprime II random prime labelling system (GE Healthcare, Amersham, UK) according to manufacturer's instructions. The membranes were soaked in 6× SSC and pre-hybridized in Shake 'n' Stack hybridization over (Hybaid, Thermo Scientific, Canada) for 4 hours at 65°C with 6 ml Church & Gilbert hybridization buffer (0.5 M NaPi, 1 mM EDTA, 7% SDS) containing 100 μg/ml heat-denatured salmon sperm DNA (Invitrogen, Life Technologies, France). Labeled genomic DNA was denatured at 100°C for 5 min and hybridization was performed during 15–18 h at 65°C in the Church & Gilbert buffer. After hybridization, each nylon membrane was washed three times in the same solution (40 mM NaPi, 1 mM EDTA, 1% SDS) for 45 min at 65°C. The membranes were then exposed to a PhosphorImager screen for 48 h. The PhosphorImager screens were scanned on a Storm 860 PhosphorImager device (GE Healthcare, Life Science, UK) at a pixel size of 50 μm. Before being re-hybridized, filters were stripped in 125 ml of a bowled buffer (10 mM Tris-HCl pH 7.6, 1 mM EDTA pH 8.0, 1% SDS) for 25 min at 100°C. A unique hybridization experiment was performed for each isolate investigated in this study and at least two independently experiments were performed for *E. coli *control strains.

### Macroarray quality control

This macroarray was first tested using the three sequenced strains, i.e. *E. coli *strain 536, strain CFT073 and *E. coli *K-12. This experiment was performed twice for strain 536, four times for strain CFT073 and three times with *E. coli *K-12 MG1655. False negative results were obtained in less than 0.01%. The average number of false positive was 8%, 9% and 12.5% using strain 536, CFT073 and K-12 MG1655 *E. coli*, respectively. Thirty-five amplicons, giving repeatedly false positive or negative results, were removed from the final analysis and are labeled as such in Additional file 1. The remaining sequences on the array were annotated as coding for (i) cell structure membrane proteins (127), (ii) putative functional enzymes involved in metabolic pathways (493), (iii) putative transcriptional regulators (63), (iv) known virulence factors (204), (v) hypothetical proteins (880), and (vi) mobile genetic elements such as transposases, IS or phages (219). To get insight into the specific function of RS218-derived sequences, we used the complete genome sequence (ColiScope project) of the phylogenetically related strain S88 [34].

### Macroarray data submission

Macroarray data were submitted to the ArrayExpress database under accession number: A-MEXP-1451.

### Macroarray data analysis

The macroarray data were analysed using the ArrayVision software (Imaging Reseach, St Catharines, Canada) for signal quantification. To avoid empirical determination of cut-offs, we developed R scripts (based on the Package Mclust for Normal Mixture modeling) adapted from a program developed for comparative genomic hybridization analysis of *Enterococcus faecalis *[63]. This analysis was based on bimodal distribution of hybridization signals, thus highlighting the existence of two different gene populations corresponding to high or low intensities. Each population of gene fits a Gaussian distribution model and allows the use of an algorithm for gene classification based on the probability that each value of probe belonged to the population of high or low hybridization signals (i.e. present, class 1 or absent genes, class -1). Parameters of the model are estimated by maximum likelihood using an expectation maximization (EM) algorithm running from the median of negative controls to the median of positive controls. Using this mathematic model, genes were classified to be either present (probability of belonging to class 1 > 80%) or absent (probability of belonging to class 1 < 20%). Some genes were considered ambiguous (probability of belonging to class 1 between 20% and 80%). R analysis was done for all the hybridized arrays and a matrix of 1 (gene present in this strain), 0 (ambiguous gene) and -1 (gene absent is this strain) was obtained. TMeV software was used with this matrix for data clustering and template matching analysis [64].

### Statistical analysis

Comparisons were based on Chi-square test for categorical variables and Fisher exact test when numbers were below five. All tests were two-tailed and p < 0.05 was considered significant. Multiple logistic regressions were used to assess the relationships between two clonal complexes, the binary variables, and explanatory variables: host characteristics and bacterial determinants. Variables with a p value less than 0.25 in univariate analyses were included into the model and interaction between significant variable was estimated. Only significant variables in the model were conserved to determine the statistically significant relationships between CC and clinical determinants. STATA version 8 was the statistical software used for Chi-square and multiple logistic regression models.

## Abbreviations

MLST: Multilocus sequence typing; ECOR: Escherichia coli reference collection; ExPEC: Extraintestinal pathogenic *Escherichia coli*; CGH: comparative genomic hybridization; CC: clonal complex; VF: virulence factor; ST: sequence type; MStree: minimum spanning tree.

## Authors' contributions

FJ, LL, OL, BP, XN and SB designed the study. VP, LD, EC and OC conceived the experiments, and gathered data and isolates. FJ, LL, VP, LD, EF, GG, BP, XN and SB analyzed the data. FJ, LL, LD, GG, OL, ED, BP, XN and SB wrote the manuscript. All authors read and approved the final manuscript.

## Supplementary Material

Additional file 1 **Clinical and microbiological features of the 161 *E. coli *bacteremia isolates.** This table gives source information and obtained results for each study strain. Click here for file

Additional file 2 **Characteristics of the DNA fragments spotted on the *E. coli *macroarray.** This file provides the characteristics (sequence, gene) of the open reading frames that were spotted on the DNA array (2,330 rows). Click here for file

Additional file 3 **List of open reading frames (ORFs) that were positively or negatively associated with B2-CC1 and/or B2-CC4, as compared to other B2 strains.** This table gives the gene name and annotated putative function of the ORFs, which presence based on DNA array results was either positively or negatively associated with B2-CC1 and/or B2-CC4, as compared to other B2 strains. This table is complementary to Figure 3. Click here for file

Additional file 4 **Correspondence of sequence type (ST) numbers between Institut Pasteur's MLST scheme and Mark Achtman's MLST scheme.** This table lists the ECOR reference strains and their ST number in both MLST schemes. Click here for file
